# Phenolic Compounds Isolated from *Caesalpinia coriaria* Induce S and G2/M Phase Cell Cycle Arrest Differentially and Trigger Cell Death by Interfering with Microtubule Dynamics in Cancer Cell Lines

**DOI:** 10.3390/molecules22040666

**Published:** 2017-04-22

**Authors:** Jessica Nayelli Sánchez-Carranza, Laura Alvarez, Silvia Marquina-Bahena, Enrique Salas-Vidal, Verónica Cuevas, Elizabeth W. Jiménez, Rafael A. Veloz G., Maelle Carraz, Leticia González-Maya

**Affiliations:** 1 Facultad de Farmacia, Universidad Autónoma del Estado de Morelos, Av. Universidad 1001, Col. Chamilpa, C.P. 62209 Cuernavaca, Mexico; jes_chazaq@hotmail.com (J.N.S.-C.); vero_7cd89@hotmail.com (V.C.); sumasr@hotmail.com (E.W.J.); 2 Centro de Investigaciones Químicas-IICBA, Universidad Autónoma del Estado de Morelos, Av. Universidad 1001, Col. Chamilpa, C.P. 62209 Cuernavaca, Mexico; lalvarez@uaem.mx (L.A.); smarquina@uaem.mx (S.M.-B.); 3 Departamento de Genética del Desarrollo y Fisiología Molecular, Instituto de Biotecnología, Universidad Nacional Autónoma de México, Cuernavaca, C.P. 62209 Morelos, Mexico; esalas@ibt.unam.mx; 4 Departamento de Ingenieria Agroindustrial, Universidad de Guanajuato, Salvatierra, C.P. 38000 Guanajuato, Mexico; alejandroveloz@hotmail.com; 5 UMR152 Pharma Dev, Université de Toulouse, IRD, UPS, 31062 Toulouse, France

**Keywords:** phenolic compounds, *Caesalpinia coriaria*, ethyl gallate, gallic acid, tannic acid, cell cycle, cancer, cell death, apoptosis, microtubules

## Abstract

*Caesalpinia coriaria* (*C. coriaria*), also named cascalote, has been known traditionally in México for having cicatrizing and inflammatory properties. Phytochemical reports on *Caesalpinia* species have identified a high content of phenolic compounds and shown antineoplastic effects against cancer cells. The aim of this study was to isolate and identify the active compounds of a water:acetone:ethanol (WAE) extract of *C. coriaria* pods and characterize their cytotoxic effect and cell death induction in different cancer cell lines. The compounds isolated and identified by chromatography and spectroscopic analysis were stigmasterol, ethyl gallate and gallic acid. Cytotoxic assays on cancer cells showed different ranges of activities. A differential effect on cell cycle progression was observed by flow cytometry. In particular, ethyl gallate and tannic acid induced G2/M phase cell cycle arrest and showed interesting effect on microtubule stabilization in Hep3B cells observed by immunofluorescence. The induction of apoptosis was characterized by morphological characteristic changes, and was supported by increases in the ratio of Bax/Bcl-2 expression and activation of caspase 3/7. This work constitutes the first phytochemical and cytotoxic study of *C. coriaria* and showed the action of its phenolic constituents on cell cycle, cell death and microtubules organization.

## 1. Introduction

Cancer can be defined as the abnormal uncontrolled proliferation of cells in the body with the ability to invade and damage other surrounding tissues. Worldwide it is one of the main causes of death [1]. It is well known that polyphenols are one of the greatest groups of phytochemicals present in plants, and one of their principal functions is to protect them from stress induced by reactive oxygen species. Polyphenols are also an essential part of the human diet, particularly flavonoids and phenolic acids which are the most common in food. There is a growing awareness that the lower incidence of cancer in certain populations may be due to the consumption of certain nutrients, especially diets rich in polyphenols. Consequently, it has been demonstrated that dietary polyphenols possess cancer preventive and therapeutic activities [2,3]. Polyphenolic compounds may promote apoptosis in cancer cells, modulating key elements in signal transduction pathways linked to apoptosis [4].

*C. coriaria* (Jack) Willd, belongs to the Caesalpinaceae family, and it is known in México with the vernacular name “cascalote” (in Náhuatl the pod-like fruit of the tree is named nacascolotl, which means twisted ear). Due to its astringent, antiseptic and anti-inflammatory properties the infusion made with the *C. coriaria* pods is traditionally used for infectious skin problems [5].

Phytochemical reports on *Caesalpinia* species have identified a high content of phenolic compounds and many studies have shown a chemopreventive or antineoplastic effect of these compounds against cancer. For example, an extract rich in polyphenols from *Caesalpinia mimosoides* Lamk showed inhibition of proliferation and induction of apoptosis in HeLa, SiHa, and C33A human cervical carcinoma cells [6]. Phenolic compounds isolated from this species have shown antiproliferative activities in cancer cells. For instance, gallic acid (GA) induced apoptosis in cholangiocarcinoma cell lines [7]) while ethyl gallate potently inhibited proliferation and induced apoptosis in MDA-MB-231 (ER-) breast cancer [8]. Another phenolic compound from *Caesalpinia* species, tannic acid (TA), was studied for its radical scavenging properties and its antitumoral effect. Recently, TA had been proven to inhibit intracellular FAS activity, down-regulating fatty acid synthase (FAS) expression in human breast cancer MDA-MB-231 and MCF-7 cells, and to induce cancer cell apoptosis [9].

Microtubules, essential constituents of the cytoskeleton in eukaryotic cells, are involved in a number of important structural and regulatory functions, including the maintenance of cell shape, intracellular transport machinery, as well as cell-growth and division [10,11]. Microtubules are in dynamic equilibrium with tubulin dimers as tubulin is polymerized into microtubules and depolymerized as free tubulin. This dynamic equilibrium is targeted by microtubule disrupting agents; often promote G2/M phase arrest of cell cycle [12]. There are several natural products like paclitaxel, podophyllotoxin, vinca alkaloids (vincristine and vinblastine), combretastatins, dolastatins, epothilones, etc. targeting microtubule dynamics. These agents either stabilize or destabilize the polymerization process of tubulins into microtubules. In both cases the equilibrium of this process is disturbed which ultimately induce cell death, therefore these natural products are used in the therapy against cancer [13].

In the present study, we have in on the one hand investigated the chemical composition of *C. coriaria* species pods, and in the other hand, we have conducted biological evaluations of the WAE extract of *C. coriaria* pods and characterized compounds on cancer cell lines with high cancer incidence and mortality as PC3 (prostate), HeLa and Ca Ski (cervical), Hep3B and HepG2 (hepatocellular) carcinoma cells. In order to explore the possible mechanism of action of these compounds, experiments were performed to determine their effects on cell cycle progression, microtubule polymerization, and cell death in the different tumor cell lines.

## 2. Results

### 2.1. Antiproliferative Activity of WAE Extract of C. coriaria

The WAE extract of the pods of *C. coriaria* was assayed to determine its antiproliferative activity against PC3 (prostate), Hep3B, and HepG2 (hepatocellular), Ca Ski and HeLa (cervical) human cancer cell lines, as well as the immortalized human hepatocytes cell line IHH as control. The corresponding IC_50_ were calculated, and the results showed that hepatocellular carcinoma HepG2 (16.5 μg/mL) and Hep3B (20 μg/mL) cells were the most sensitive to the WAE extract, while IHH cells, which are not tumor cells (202 μg/mL), were the less sensitive (Table 1). The WAE extract of *C. coriaria* showed also antiproliferative activities on the PC3, Ca Ski and HeLa cells with IC_50_ values of 24, 25.3 and 40 μg/mL, respectively. These results were consistent enough to envisage the fractionation and isolation of the active compounds.

### 2.2. Isolation of Active Compounds 

Next the WAE extract of *C. coriaria* was fractionated by repeated column chromatography to obtain stigmasterol, ethyl gallate (EG), and GA. Stigmasterol was identified by direct comparison with the spectroscopic data of an authentic sample, while EG and GA were identified and characterized by spectroscopic data analysis and comparison with data described in the literature.

The ^1^H-NMR spectrum of GA showed only two single signals at δ 13.5 (1H, s), and δ 7.14 (2H, s) indicating the presence of benzoic acid (Figure S2). The ^13^C-NMR spectrum shows a signal at δ 168.25 characteristic of the carbon atom of a carboxylic acid group, as well as four aromatic carbon signals at δ 146.0 (2 C), 138.8 (1 C), 122.0 (1 C), and 110.2 (2 CH), indicating the presence of a symmetrical tetrasubstituted ring (Figure S3). Comparison of these spectral data, together with the mass spectrum (Figure S1) with those described for the same compound in the literature confirmed its structure [14].

**Figure 1:**
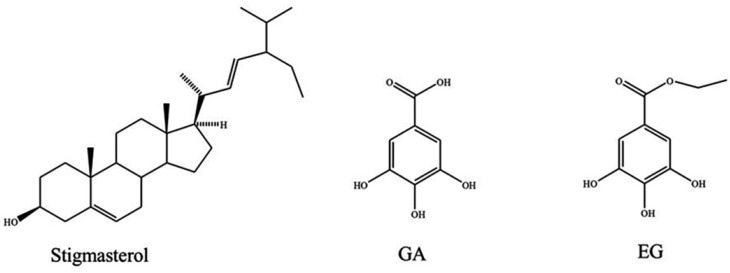
Compounds isolated from the WAE extract of *C. coriaria*.

The ^1^H-NMR spectrum of EG displayed signals for a phenol at δ 8.2 (1H, sbr), one tetrasubstituted aromatic ring at δ 7.12 (2H, s), and one ethoxy group at δ 4.24 (2H, q, *J* = 7 Hz) and δ 1.30 (3H, t, *J* = 7 Hz) (Figure S5). The ^13^C-NMR showed the same signals as GA, except for the presence of the signals for the ethoxy group at δ 60.96 and 14.69 (Figure S6). The above spectral features are in close agreement to those observed for EG (Figure 1) [14]. The most polar extract was constituted mainly of tannic acid, which was previously identified in this species [15]. For further biological studies, we didn’t purify this substance from the WAE extract but rather used a commercial sample.

### 2.3. Antiproliferative Activity of Pure Compounds

Antiproliferative activities evaluation of the isolated compounds, GA, EG, stigmasterol, and TA, showed interesting effects on cell proliferation (Table 1). GA inhibited cell proliferation in all cancer cell lines analyzed, but was most potent on hepatocellular carcinoma cells with IC_50_ of 35.8 µM and 46.4 µM on HepG2 and Hep3B cells, respectively. Interestingly the IC_50_ of GA on IHH immortalized human hepatocytes cells was much higher (146 µM). Similar results were observed with EG exhibiting the best antiproliferative activity on Hep3B cells (IC_50_ of 38 µM) and the worse on IHH cells (IC_50_ of 211µM). With respect to TA, the most sensitive cells were Hep3B (IC_50_ of 11 µM), followed by PC3 (IC_50_ of 12.9 µM) and Ca Ski cells (IC_50_ of 13 µM). Finally, we found that on all the cancer cell lines tested, stigmasterol was not active, with an IC_50_ higher than 90 µM.

### 2.4. Cell Cycle Analysis

**Figure 2:**
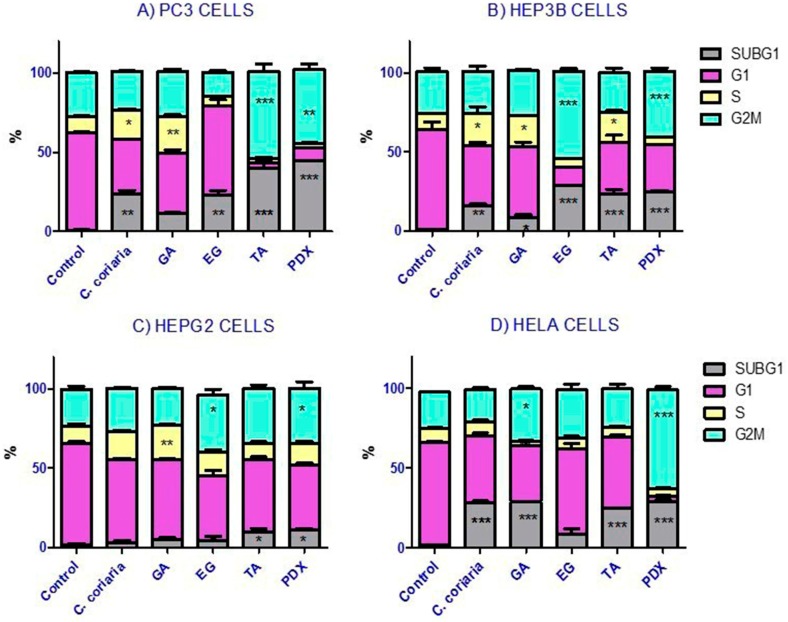

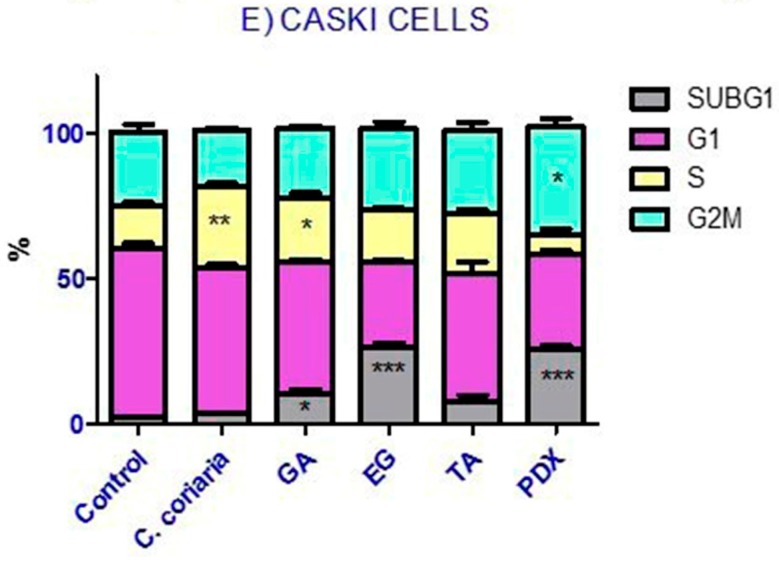
Effect of the WAE extract of *C. coriaria*, GA, EG and TA on cell cycle progression in cancer cells lines. (**A**) PC3 (prostate); (**B**) Hep3B and (**C**) HepG2 (hepatocellular carcinoma); (**D**) HeLa and 2E) Caski (cervical cancer). Podophillotoxin (PDX) was used as a positive control (0.005 µM). * *p* < 0.05, ** *p* < 0.01, *** *p* < 0.001 compared with the control group.

The effect of the WAE extract and the isolated compounds of C. coriaria on cell cycle progression of cancer cells was determined at the corresponding IC_50_. Podophyllotoxin (PDX) was included as a positive control of the G2/M phase arrest. Histograms acquired are shown in Supplementary Materials in the following order, PC3 (Figure S7); Hep3B (Figure S8); HepG2 (Figure S9); Hela (Figure S10) and CaS ki (Figure S11) cells. Interestingly, the different compounds showed differential effects on the cell cycle progression of the different cell lines (Figure 2). More specifically, we observed that the WAE extract induced an S phase arrest in PC3 (Figure 2A), Hep3B (Figure 2B) and Ca Ski cells (Figure 2E). Moreover GA induced an S phase arrest too in PC3 (Figure 2A), Hep3B (Figure 2B) and HepG2 cells (Figure 2C). TA induced an S phase arrest only in Hep3B cells (Figure 2B). On the other hand, the characterized compounds, but not the extract of C. coriaria, induced a G2/M phase cell cycle arrest depending on the cells observed. GA induced a G2/M phase arrest in HeLa cells (Figure 2D), while TA increased the population of G2/M phase of PC3 cells (Figure 2A). EG increased meaningfully the G2/M population in Hep3B cells (Figure 2B) and in HepG2 cells (Figure 2C). After treatment with the WAE extract, EG, GA, and TA, most of cancer cells showed a significant increase in the subG1 population (Figure 2A–E). This suggests DNA damage, cell death or cellular senescence. PC3 and Hep3B cells were the most affected (Figure 2A,B), followed by HeLa cells (Figure 2D) and Ca Ski cells (Figure 2E). That was also the case in HepG2 cells treated with EG or TA (Figure 2C).

### 2.5. Effect of Characterized Compounds on Microtubules Dynamics

**Figure 3:**
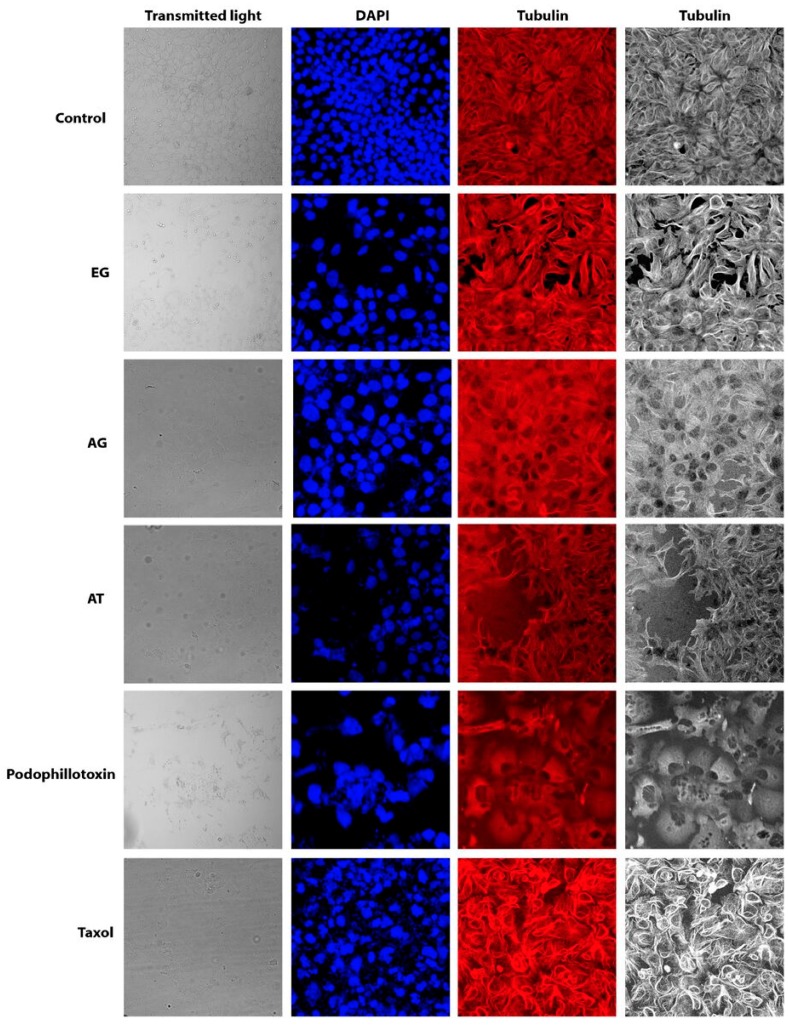
Effect of isolated compounds on stabilization of microtubules in Hep3B cells by immunofluorescence of α-Tubulin and confocal microscopy. EG; GA; TA; Podophillotoxin (Microtubules destabilizing agent) and Taxol (Microtubules stabilizing agent).

With the purpose of exploring the effect on microtubule dynamics, immunofluorescence of α-tubulin in Hep3B cells in the presence of pure compounds EG, GA, and TA was monitored by confocal microscopy. Hep3B cells had already been reported as a good cellular model to analyze microtubule perturbations because of their phenotypic characteristics [16]. Taxol and podophyllotoxin, which are known to target microtubules, were used as positive controls. As expected, we obtained a complete disassembly of microtubules after podophyllotoxin treatment and a stabilization of microtubules by an inhibition of their depolymerization after taxol treatment (Figure 3). Our results showed that in Hep3B cells treated with EG the microtubules appeared rigid and formed large bundles in the cytoplasm. EG induced a complete rearrangement of microtubules structure. It is clear that polymerized microtubules formed bulky rigid structures, localized in the cell periphery, in a manner parallel to the plasma membrane. We observed that they are typically arranged perpendicular to the cell edge and the cells showed microtubules stabilized around the nucleus, similar but to a lesser extent than observed in taxol treated cells. These structures were also observed in the cells treated with tannic acid, despite the low fluorescence intensity detected. These results are in accordance with those of the cell cycle analysis suggesting that EG could induce cell cycle G2/M phase arrest in hepatocellular carcinoma cells by stabilizing microtubule polymerization. This is the first report describing the effect of EG on microtubule stabilization in cancer cells. Moreover GA treated cells showed a disperse tubulin signal although some microtubules appear normal, indicating a mild effect of GA on microtubule stability (Figure 3). Moreover, we evaluated the effect of EG on PC3 cells and although some polymerized microtubules localized in the cell periphery were observed, the changes observed were not significant respect to taxol treatment in this cell line (Figure S12). Further studies with these compounds would be interesting to further determine their action mechanism on microtubules and a possible interaction with tubulins.

### 2.6. Cellular Death 

**Figure 4:**
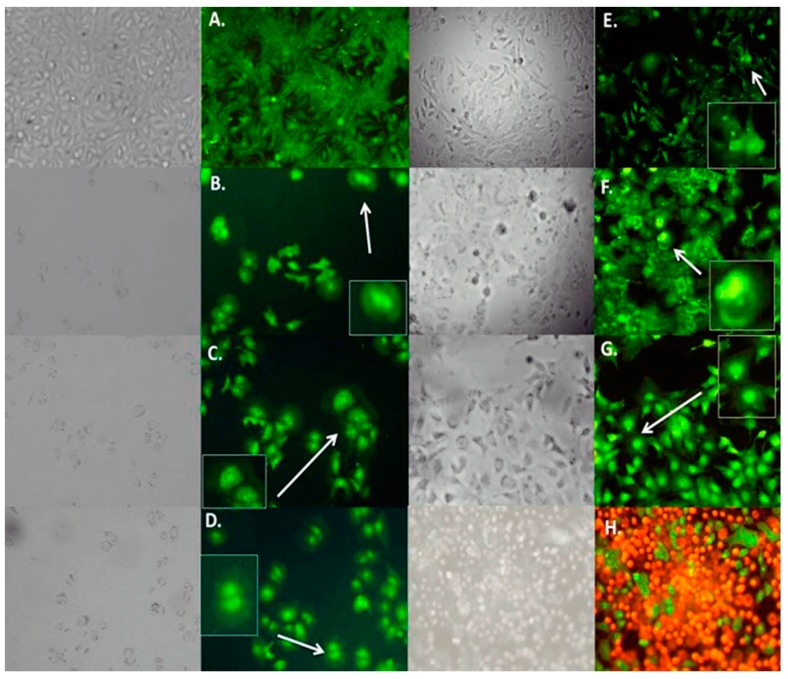
Effect of WAE extract of *C. coriaria* extract and pure compounds on cell death in Hep3B cells by epifluorescence microscopy. (**A**) Negative control; (**B**) *C. coriaria* extract; (**C**) GA; (**D**) EG; (**E**) TA; (**F**) Podophillotoxin 0.005 µM (positive control); (**G**) H_2_O_2_ apoptosis positive control; (**H**) Necrosis control.

Apoptosis is a central mechanism to eliminate unwanted cells that may accumulate during physiological processes and pathologic conditions such as cancer and autoimmune diseases. The induction of apoptosis promotes morphological characteristic changes: cell shrinkage (cells get smaller in size, the cytoplasm is dense and the organelles are more tightly packed), pyknosis which is the result of chromatin condensation and this is the most characteristic feature of apoptosis, extensive plasma membrane blebbing karyorrhexis and separation of cell fragments into apoptotic bodies during a process called “budding”. Apoptotic bodies consist of cytoplasm with tightly packed organelles with or without a nuclear fragment [17]. In the present study, the effect of the *C. coriaria* extract and pure compounds (AG, AG and TA) on cell death was characterized by morphological changes observed by acridine orange-ethidium bromide fluorescence microscopy. Hep3B (Figure 4), PC3 (Figure S13), and HepG2 (Figure S14) cells showed morphological characteristic changes similar to the pro-apoptotic controls podophyllotoxin and H_2_O_2_. For instance, Hep3B cells treated with the *C. coriaria* extract, AG and EG showed clear chromatin condensation and plasma membrane blebbing (magnification, white arrows Figure 4B–D); while the formation of apoptotic bodies and chromatin condensation was observed after the TA treatment (magnification, white arrows 4E); the morphological changes were similar to podophyllotoxin (Figure 4G). Major effects on cell death in PC3, HepG2 and CaSki cells were observed after TA treatment with a clear chromatin condensation, plasma membrane blebbing and formation of apoptotic bodies (white arrows Figure S13E, S14E and S15E, respectively). Similar results were observed in PC3 cells after the *C. coriaria* extract treatment (Figure S13B). On HeLa cells, chromatin condensation and green yellow cores (white arrows) were observed only with GA treatment (Figure S16D). It is noteworthy that necrosis was not observed in none of the cells treated with the compounds. These results showed that the WAE extract of *C. coriaria* as well as the isolated molecules EG, GA and TA induced cell death, with characteristic apoptotic morphological changes, that are in accordance with the increase of subG1 population previously observed in the cell cycle histograms (Figures S7–S11) and bar charts (Figure 2).

### 2.7. Bax and Bcl2 Expression

**Figure 5:**
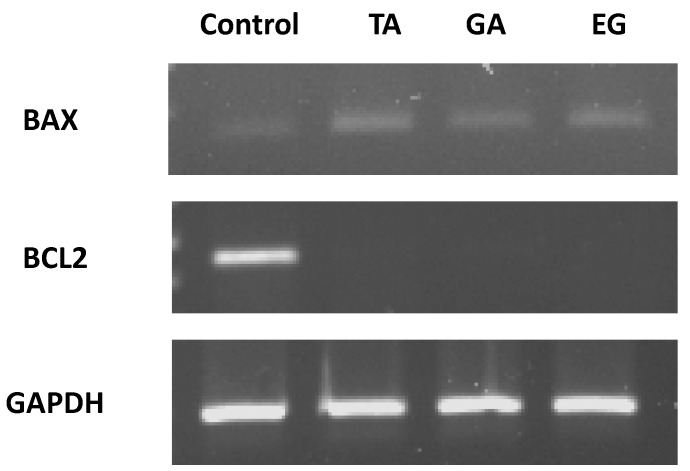
Effect of TA, GA and EG on mRNA expression levels of Bcl-2 and Bax in Hep3B cells after 72 h treatment. *GAPDH* was used as an internal control.

In order to determine whether the pure compounds induce cell death by apoptosis by regulating anti-apoptotic (Bcl-2) and pro-apoptotic (Bax) proteins in the hepatocellular carcinoma Hep3B cells, expression of Bax and Bcl-2 was analyzed by RT-PCR. Ours results showed a clear inhibition of Bcl-2 and an increase of Bax transcripts after the treatment with TA, GA and EG (Figure 5). The Bax/Bcl-2 ratio was evidently increased when compared to the control after TA, GA and EG treatments.

### 2.8. Caspases 3/7 Activity

**Figure 6:**
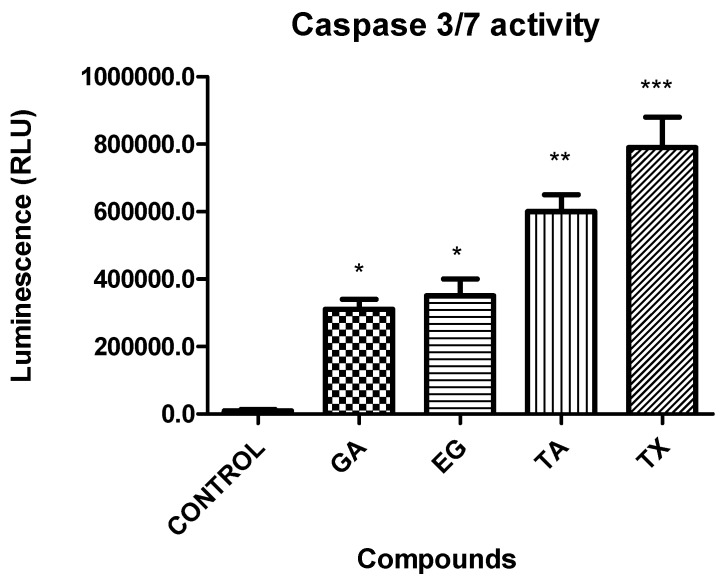
Caspase 3/7 activity after treatment with GA; EG; TA and taxol (TX) in Hep3B cells. * *p* < 0.05, ** *p* < 0.01, *** *p* < 0.001 were obtained when compared to the negative control.

Caspases play a key role in the initiation and execution of apoptosis. Therefore to support the effect previously observed on Bax/Bcl-2 expression; the caspase 3/7 activity was evaluated on Hep3B cells after treatment by GA, EG and TA (Figure 6). All pure compounds showed a higher caspase 3/7 activity when compared to the non-treated control. TA showed the most significant effect, just below taxol (positive control).

## 3. Discussion

A number of studies have demonstrated the antitumoral and antimutagenic properties of polyphenols and phenolic compounds isolated from plants [15]. Our results with the WAE extract of *C. coriaria* and isolated phenolic compounds on cell proliferation, cell cycle arrest and cell death confirm such antitumoral potential. In this study, the hepatocellular carcinoma cells were the most sensitive to the antiproliferative effect of the WAE extract of *C. coriaria*, although they are known to be chemoresistant. Oppositely, the immortalized cells from normal human hepatocytes (IHH cells), were the less sensitive to the WAE extract of *C. coriaria* extract and compounds with an IC_50_ 10 fold higher than the one seen for Hep3B cancer cells. This effect was expected, as it seems that this extract showed selectivity for cancer cells. Moreover, the WAE extract of *C. coriaria* induced S phase cell cycle arrest in PC3, Hep3B and CaSki cells but no changes were observed in G2/M population cells. Previous studies with extracts rich in phenolic compounds have shown S phase cell cycle arrest as mechanism of inhibition of cancer cell proliferation [18]. In addition, we observed a significant increase in the subG1 population (in the cell cycle analysis) by the WAE extract of *C. coriaria* treatment in PC3 and Hep3B cells that correlated with the observed morphological characteristic changes similar to apoptosis.

Further chromatographic fractionation of the WAE extract of *C. coriaria* allowed the isolation or identification of stigmasterol, EG, GA and TA. GA and TA had been previously identified as components of the WAE extract of *C. coriaria* but the presence of stigmasterol and EG had not been reported before in this species [15]. We found that GA, a metabolic intermediate of plants seemed to act selectively on cancer cells, particularity against hepatocellular carcinoma cell lines with IC_50_ values 4- and 3-fold lower in HepG2 and Hep3B cells, respectively, compared to the one in normal IHH cells. This selectivity had been also observed in dRLh-84 mouse hepatoma cells that showed cell death after treatment with GA at a concentration of 20 μg/mL, while in primary cultures of rat hepatocytes and macrophages it was not cytotoxic, showing again some selectivity for cancer cells [19]. EG is an ester derivative of GA that we also found more anti-proliferative on Hep3B and HepG2 cells than on IHH cells (with a 5- and 3-fold lower IC_50_, respectively). Finally, TA, known as a potent anti-oxidant, showed lower IC_50_ values than EG and GA in the majority of the cancer cells.

Interestingly, GA induced cell cycle arrest differentially in PC3, Hep3B and HepG2 cells, where we observed an S phase arrest, than in HeLa cells where we observed a G2/M phase arrest. Previously, it was demonstrated that GA treatment of human prostate carcinoma DU145 cells resulted in an S phase cell cycle arrest [20]. Recently, it was elucidated that GA induced G2/M phase arrest in HeLa cells accompanied by mitotic catastrophe, and formation of cells with multiple nuclei, followed by impaired centrosomal clustering [21]. Similarly, TA induced S phase arrest in Hep3B whereas it induced a G2/M phase arrest in HepG2 and PC3 cells. A preceding study in malignant human cholangiocytes indicated for TA an S phase cell cycle arrest [22]. Finally EG induced a clear G2/M phase cell cycle arrest in the hepatocellular carcinoma Hep3B and HepG2 cells. One explanation of a cell cycle G2/M phase arrest is the inhibition by stabilization or destabilization of microtubules polymerization as that can be observed with taxol or podophillotoxin. Our results showed by confocal microscopy a microtubules stabilization of Hep3B cells treated with EG. Similar results but to a lesser extent were observed with TA. Interestingly we expected to observe an effect on microtubules destabilization rather than stabilization since it has been previously shown that the 3,4,5-trimethoxyphenyl unit of GA, present in the EG structure, is crucial for interactions disturbing the assembly of tubulin [23,24]. Therefore, further studies on the activity of EG on microtubule dynamics in vitro are necessary to better understand the EG mechanism of action.

It was known that GA induces apoptosis by activating PARP, caspase-9, and caspase-3, related to an increase of the pro-apoptotic Bax expression [25]. In addition, TA was known to induce apoptotic death in acute myeloid leukemia (AML) HL-60 cells in a dose- and time-dependent manner as well as an increase of the sub-G1 fraction, chromosome condensation, and DNA fragmentation [26]. EG was reported to induce DNA fragmentation and apoptosis via mitochondrial-mediated pathways by increasing the ratio of Bax/Bcl-2 and activation of Caspases-8, -9, -3 in HL-60 cells [27]. Similar results were given when EG was tested against the human breast cancer cells (MDA-MB-231 (ER-) invasion and showed to modulate the PI3K/Akt pathway and to inhibit their downstream targets such as Bcl-2/Bax at mRNA levels [8]. This motivated us to look at the effect of the GA, TA and EG compounds on apoptosis on Hep3B hepatocarcinoma cells. We found chromatin condensation, plasma membrane blebbing and formation of apoptotic bodies. Moreover, GA, EG and AT clearly inhibited Bcl-2 and increased Bax transcripts in Hep3B cells and showed a clear increase in caspase 3/7 activation. This is in line with a pro-apoptotic activity of these compounds in Hep3B cells.

## 4. Materials and Methods

### 4.1. Plant Material

The pods of *C. coriaria* were collected by Jessica Nayelli Sánchez Carranza on March 2013 in Cutzamala the Pinzon Guerrerro, México. A sample was authentified by Alejandro Flores and deposited at the Herbarium of the Universidad Autónoma del Estado de Morelos (HUMO-UAEM), with the voucher number 33400.

### 4.2. Chemical Procedures

Isolation procedures and purity of compounds were checked by thin layer chromatography (TLC, Merck Millipore, Billerica, MA, USA) visualized by UV light, and sprayed with Ce(SO_4_)_2_·2(NH_4_)_2_SO_4_·2H_2_O. All ^1^H, ^13^C, and 2D-NMR experiments were recorded on acetone-*d*_6_, on a Varian Unity 400 spectrometer (Varian, Palo Alto, CA, USA), at 400 MHz for ^1^H-NMR, and at 100 MHz for ^13^C-NMR. EI-EM spectra were recorded on a JEOL JMX-AX 505 HA mass spectrometer (Jeol Ltd., Tokyo, Japan). All the reagents and solvents used were of analytical grade. Tannic acid (CAS: 1401-55-4) was purchased from Sigma Aldrich (St. Louis, MO, USA).

### 4.3. Extraction and Isolation.

The collected material (600 g) was dried and powdered and extracted with 2 L of a water:acetone:ethanol solvent mixture (80:10:10, *v*/*v*) at room temperature. After 72 h, the extract was filtered and concentrated under reduced pressure at 60 °C, to yield 75 g of extract. This WAE extract of *C. coriaria* was dissolved in acetone and filtered again. The soluble part (12.4 g) was subjected to a first chromatographic fractionation using Si-gel open column chromatography (60 × 10 cm) and eluted with a gradient of CH_2_Cl_2_–acetone (from CH_2_Cl_2_ 100% to CH_2_Cl_2_–acetone 70:30, 200 mL each fraction) to obtain three main fractions JS33A, 1.3 g (100:0); JS33B, 900 mg (90:10); JS33C, 2.4 g (80:20). Fraction JS33A was subjected to CC purification to yield 72 mg of stigmasterol. Fraction JS33B was purified by column chromatography (30 g Si gel), eluted with a gradient of *n*-hexane–acetone. Fractions eluted with *n*-hexane–acetone (75:25) contained pure ethyl gallate, which was crystallized from CH_2_Cl_2_–EtOH, to yield 820 mg of ethyl gallate. Fraction JS33C was purified by column chromatography eluted with a CH_2_Cl_2_:acetone gradient system (100:00→80:20). Fractions eluted with CH_2_Cl_2_–acetone (62:38) afforded 920 mg of a yellow solid, which was purified by column chromatography using an isocratic mixture of CH_2_Cl_2_–MeOH to yield 501.4 mg of gallic acid. These compounds were characterized by comparison of their spectroscopic data with those described [14].

### 4.4. Cytotoxic Assay

The WAE extract of *C. coriaria* as well as pure compounds **1** and **2** and tannic acid (**3**) were subjected to antiproliferative assays in PC3 (prostate), Hep3B, and HepG2 (hepatocellular), and Ca Ski and HeLa (cervical) human cancer cell lines, obtained from ATCC (American Type Culture Collection, Manassas, VA, USA). We also included an immortalized human hepatocytes cell line (IHH) as a control of non-cancerous cells [28]. PC3 and CaSKi cells were grown in RPMI-1640 medium (Sigma Aldrich, St. Louis, MO, USA), while Hep3B, HepG2, IHH and HeLa in DMEM medium (Invitrogen, Thermo Fisher Scientific, Inc., Waltham, MA, USA) and supplemented with fetal bovine serum 10% (SFB, Invitrogen) and with 2 mM glutamine, all cultures were incubated at 37 °C in atmosphere of 5% CO_2_. 4000 cells per well in 96-well plate were cultured for starting the cytotoxic evaluation. The extract was solubilized in culture medium and sterilized by 0.2 µm membrane filtration, the concentrations used for the extract were 1000, 100, 10, 1, 0.1, 0.001 μg/mL for a dose/response curve, and incubated at 37 °C in 5% CO_2_ atmosphere for 72 h. Concentrations of 160, 80, 40, 20, 10, 5, 2.5, 1.25, 0.6, 0.3 μg/mL were used for pure compounds (1–3), and incubated at 37 °C in 5% CO_2_ atmosphere for 72 h. The number of viable cells in proliferation was then determined by using CellTiter 96^®^ AQueous One Solution Cell Proliferation Assay kit (Promega, Madison, WI, USA), following the manufacturer's instruction. Cell viability was determined by absorbance at 450 nm using an automated ELISA reader. The experiments were conducted by triplicate in three independent experiments. Data were analyzed in Prism 5.0 statistical program and the IC_50_ values were determined by regression analysis.

### 4.5. Cell Cycle Analysis

PC3, Hep3B, HepG2, HeLa and Ca SKi cells (1.25 × 10^5^) were plated in 6-well plates. Exponential growing cells were exposed to each compound for 72 h in accordance with their IC_50_ values. Podophyllotoxin (PDX) at 0.005 µM was included as a positive control of the G2/M phase arrest. Cells from each treatment were trypsinized and collected into single cell suspensions, centrifuged, and fixed in cold ethanol (70%) overnight at −20 °C. The cells were then treated with RNase (0.01 M, Sigma Aldrich) and stained with propidium iodide (PI) (7.5 μg/mL, Invitrogen) for 30 min in the dark, PI has the ability to bind to DNA molecules, and then RNase was added in order to allow PI to bind directly to DNA. The percentage of cells in G1, S, and G2 phases was analyzed with a flow cytometer (Becton Dickinson, FACS Calibur, San Jose, CA, USA); the number of cells analyzed for each sample was 10,000. The experiments were conducted by triplicate in three independent experiments. Data obtained from the flow cytometer were analyzed using the FlowJo Software (Tree Star, Inc., Ashland, OR, USA) to generate DNA content frequency histograms, and to quantify the number of cells in the individual cell cycle phases. The results were summarized in bar charts arranged by cell line, compound and cell cycle phase. Data were analyzed in Prism 5.0 statistical program and statistical differences were evaluated using ANOVA followed by Dunnett test and considered significant at *p* value < 0.05.

### 4.6. Immunofluorescence of α-Tubulin

3.5 × 10^4^ Hep3B cells were added in 24-well culture plates containing glass slides and allowed to attach overnight at 37 °C in 5% CO_2_. Then they were treated with ethyl gallate (7.7 μg/mL), GA (7.9 μg/mL), tannic acid (20 μg/mL) and controls podophillotoxin (0.005 µM) and taxol (75 nM) for 72 h. The cells were fixed with paraformaldehyde (PFA, 4% in PEM buffer). After 15 min PFA/NaHCO_3_ was added and incubated for 45 min at room temperature. The slides were rinsed with PBS and treated with 0.1% Triton X-100 (Sigma Aldrich) and then incubated with the primary antibody mouse anti-α-tubulin (1:300, T9026, Sigma Aldrich) overnight at 4 °C. The secondary antibody (anti-mouse Alexa 647 1:1000, A-21235, Molecular Probes, Thermo Fisher Scientific, Inc., Waltham, MA, USA) was added and incubated for two hours at 37 °C. The cells were stained with Sytox Green (1:5000, S7020, Molecular Probes) for one hour, mounted, and imaged by confocal microscopy. The experiments were conducted by triplicate in three independent experiments.

### 4.7. Cell Death

PC3, Hep3B, HepG2, HeLa, and CaS Ki cells were cultured 20000 cells per well in 24-well plates, and then were treated for 72 h according to the IC_50_ of each compound in each cell line. For controls, cells were grown 71 h before being treated 30 min with H_2_O_2_ (positive control of apoptosis) or being boiling in water at 95 °C for 10 sec (positive control of necrosis). After 72 h of treatment cells were exposed to a solution of acridine orange (AO) and ethidium bromide (EB) (100 μg/mL AO, 100 μg/mL EB), according to procedures reported [29]. Cells were observed using a fluorescence microscope. AO/EB are fluorescent intercalating nucleic acids that when bounded to DNA give green and orange fluorescence respectively. It is well known that AO can pass through cell membranes, but EB cannot. Necrotic cells stain red but have a nuclear morphology resembling that of viable cells. Apoptotic cells appear green, and morphological changes such as formation of apoptotic bodies are observed. The criteria for identification are as follows: viable cells appear to have green nucleus with intact structure; early apoptosis cells exhibit a bright green nucleus showing condensation of chromatin; late apoptosis appears as dense orange areas of chromatin condensation; and orange intact nucleus depicts secondary necrosis [30,31]. The experiments were conducted by triplicate in three independent experiments.

### 4.8. RT-PCR

1.25 × 10^5^ Hep3B cells were plated and treated with GA, TA and EG for 72 h., then RNA was isolated. The total RNA extraction was performed employing a Quick-RNA MiniPrep Kit (Zymo Research, Irvine, CA, USA), following the manufacturer’s instructions. RNA was quantified using NanoDrop^®^ ND-1000 (Thermo Scientific, Waltham, MA, USA), and the RNA content of the samples was normalized. The RT-PCR was performed using a One-Step RT-PCR Kit with Thermo-Start Taq (Thermo Scientific) following the manufacturer’s instructions.

The primer sequences for Bcl-2 were 5′-CCC TCC AGA TAG CTC ATT-3′, and 5′-CTAGAC AGACAAGGAAAG-3′. The Bax primer sequences were 5′-ATGGACGGGTCCGGGGAG-3′, and 5′-TCAGAAAACATGTCAGCTGCC-3′ [32]. The GAPDH primers were 5′-CAAGGTCATCCA TGACAACTTTG-3′ and 5′-GTCCACCACCCTGTTGCTGTAG-3′. All primers were synthesized by IDT-Integrated DNA Technologies (Redwood, CA, USA), the reaction products of the samples were analyzed in 1.5% agarose gel.

### 4.9. Caspases Activity

Hep3B cells (8000 per well) were plated on 96-well plate and treated with GA, TA and EG for 72 h. After treatment the caspase 3/7 activity was determined using the luminescent Caspase-Glo^®^ 3/7 Assay (Promega, cat. G811C) following the manufacturer’s instructions. The results were represented as relative units of luminescence and represented in graphs, the statistical analysis was performed using the Prism 5.0 statistical program and the test performed was *t*-student test with significant *p* values < 0.05.

## 5. Conclusions

Our results suggest that the extract of *C. coriaria* and its isolated phenolic compounds exhibit strong antiproliferative activities by inducing cell cycle arrest, apoptosis, microtubules reorganization. Ethyl gallate showed an interesting effect on Hep3B hepatocellular carcinoma cells by microtubule stabilization and promoting cell cycle G2/M phase arrest and cell death. These results show the great antineoplasic potential of these phenolic compounds on cancer cells. Further studies will have to be performed to determine the precise mechanism of action of each of these phenolic compounds on the studied cancer cells. This is the first report about the antiproliferative biological activities of the species *C. coriaria*.

## Figures and Tables

**Table 1:** IC_50_ values in μg/mL for the *C. coriaria* extract and μM for isolated compounds.

Extract/Compound	Cell Lines					
	CaSki	HeLa	PC3	Hep G2	Hep3B	IHH
Extract *C. coriaria* (μg/mL)	25.3 ± 2.7	40 ± 4.0	24 ± 2.5	16 ± 2.4	20 ± 2.3	202 ± 18.0
Gallic acid (µM)	51.72 ± 8	58 ± 18	58.7 ± 7	35.8 ± 9	46.4 ± 11	146 ± 12
Ethyl gallate (µM)	68.12 ± 10	201 ± 5	60 ± 2	75 ± 6	38 ± 3	211 ± 10
Stigmasterol (µM)	ND	97 ± 9	ND	ND	90 ± 12	ND
Tannic acid (µM)	13 ± 2	22 ± 3	12.9 ± 1.8	23 ± 0.8	11 ± 1.2	24 ± 0.2

IC_50_ values represent the concentration causing 50% growth inhibition. They were determined by linear regression analysis. Each sample is the mean of three independent experiments. ± standard deviation (SD).

## Supplementary Materials

Supplementary materials are available online.
