# Supplementary material for: Phenolic Compounds Isolated from Caesalpinia coriaria Induce S and G2/M Phase Cell Cycle Arrest Differentially and Trigger Cell Death by Interfering with Microtubule Dynamics in Cancer Cell Lines
Source: Molecules. 2017 Apr 22;22(4):666. doi: 10.3390/molecules22040666 (PMC6154320; doi:10.3390/molecules22040666)
Supplement: Supplementary file 1 [file molecules-22-00666-s001.pdf]

Article

Phenolic compounds isolated from Caesalpinia coriaria induce S and G2/M phase cell cycle arrest differentially and trigger cell death by interfering with microtubule dynamics in cancer cell lines

Jessica Nayelli Sánchez Carranza [1], Laura Álvarez Berber [2], Silvia Marquina Bahena [2], Enrique Salas Vidal [3], Verónica Cuevas [1], Elizabeth W. Jimenez [1], Rafael A. Veloz G. [4], Maelle Carraz [5] and Leticia González-Maya [1]*

[1] Facultad de Farmacia, Universidad Autónoma del Estado de Morelos, Av. Universidad 1001, Col. Chamilpa, Cuernavaca, México; jes_chazaq@hotmail.com (J.N.S.); vero_7cd89@hotmail.com (V.C.); sumasr@hotmail.com (E.W.J.); letymaya@uaem.mx (L.G.M.)

[2] Centro de Investigaciones Químicas-IICBA, Universidad Autónoma del Estado de Morelos, Av. Universidad 1001, Col. Chamilpa, Cuernavaca, México; lalvarez@uaem.mx (L.A.B.); smarquina@uaem.mx (S.M.B)

[3] Departamento de Genética del Desarrollo y Fisiología Molecular de Biotecnología, Universidad Nacional Autónoma de México, Cuernavaca Morelos, México; esalas@ibt.unam.mx

[4] Departamento de Ingenieria Agroindustrial, Universidad de Guanajuato, Salvatierra, Guanajuato, México; alejandroveloz@hotmail.com

5 UMR152 PharmaDev, Université de Toulouse, IRD, UPS, France; maelle.carraz@ird.fr

* Correspondence: letymaya@uaem.mx; Tel.: +52 777 3297000, ext 2325

List of Figures

Figure S1. Mass spectrum of gallic acid

Figure S2. NMR ^{1}H spectrum of gallic acid [(400 MHz, ACETONE-d_6), δ (ppm)].

Figure S3. NMR ^{13}C spectrum of gallic acid [(100 MHz, ACETONE-d_6), δ (ppm)].

Figure S4. Mass spectrum of ethyl gallate

Figure S5. NMR ^{1}H spectrum of ethyl gallate [(400 MHz, ACETONE-d_6), δ (ppm)].

Figure S6. NMR ^{13}C spectrum of ethyl gallate [(100 MHz, ACETONE-d_6), δ (ppm)].

Figure S7. Effect of *C. coriaria* extract and isolated compounds on cell cycle in PC3 cell line of prostate cancer.

Figure S8. Effect of *C. coriaria* extract and isolated compounds on cell cycle in Hep3B cell line of hepatocellular carcinoma

Figure S9. Effect of *C. coriaria* extract and isolated compounds on cell cycle in HepG2 cell line of hepatocellular carcinoma

Figure S10. Effect of *C. coriaria* extract and isolated compounds on cell cycle in HeLa cell line of cervical cancer.

Figure S11. Effect of *C. coriaria* extract and isolated compounds on cell cycle in Ca Ski cell line of cervical cancer.

Figure S12. Effect of isolated compounds on stabilization of microtubules by confocal microscopy in PC3 cells.

Figure S13. Effect of extract *C.coriaria* and isolated compounds on cell death by epifluorescence microscopy in PC3 cell line of prostate cancer.

Figure S14. Effect of extract *C.coriaria* and isolated compounds on cell death by epifluorescence microscopy in HepG2 cell line of hepatocellular carcinoma

Figure S15. Effect of extract *C.coriaria* and isolated compounds on cell death by epifluorescence microscopy in HeLa cell line of cervical cancer.

Figure S16. Effect of extract *C.coriaria* and isolated compounds on cell death by epifluorescence microscopy in CaS ki cell line of cervical cancer.

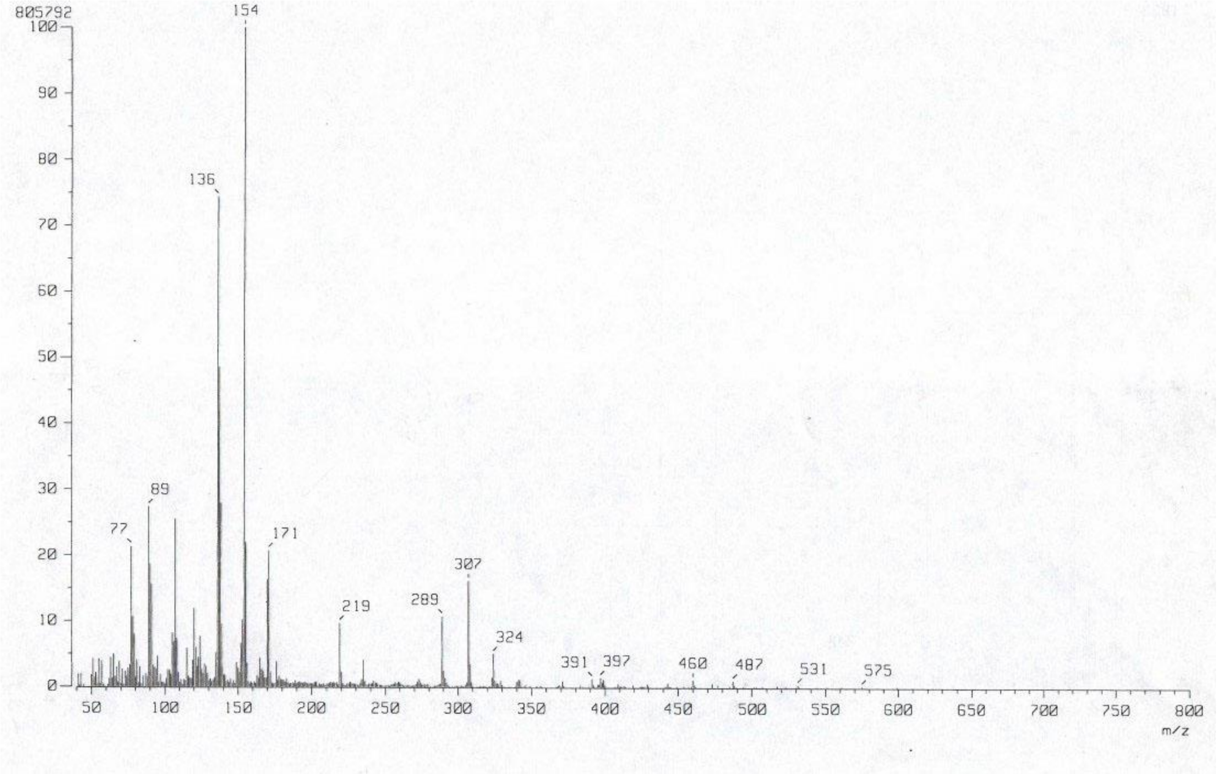

Figure S1. Mass spectrum of gallic acid

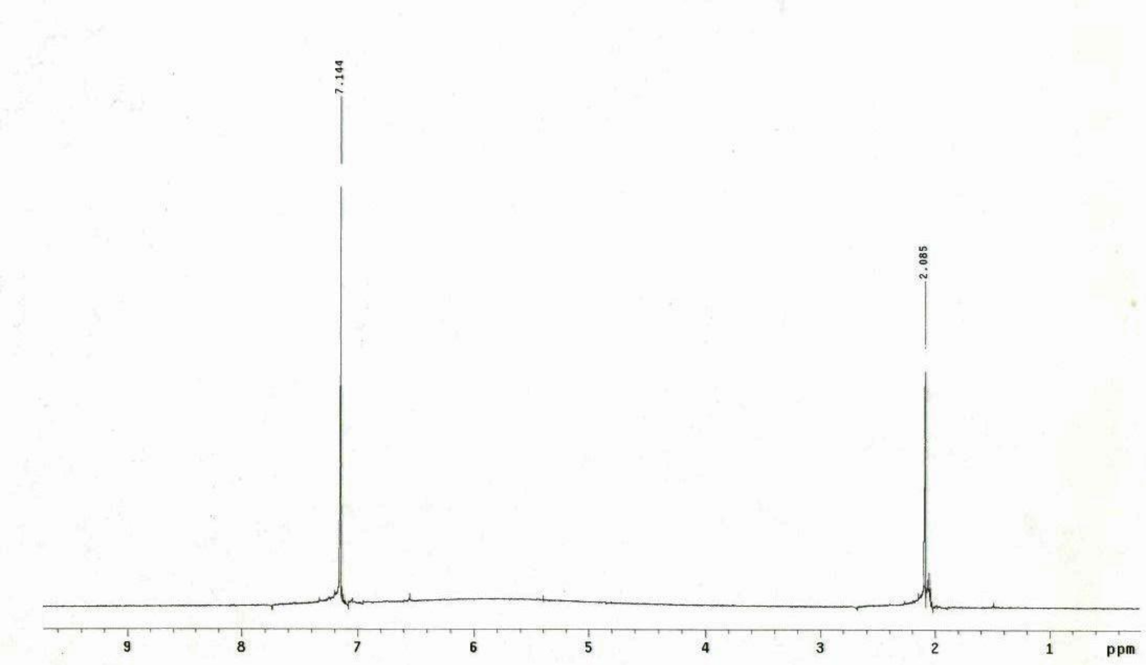

F

igure S2. NMR ^{1}H spectrum of gallic acid [(400 MHz, ACETONE-d_6), δ (ppm)]

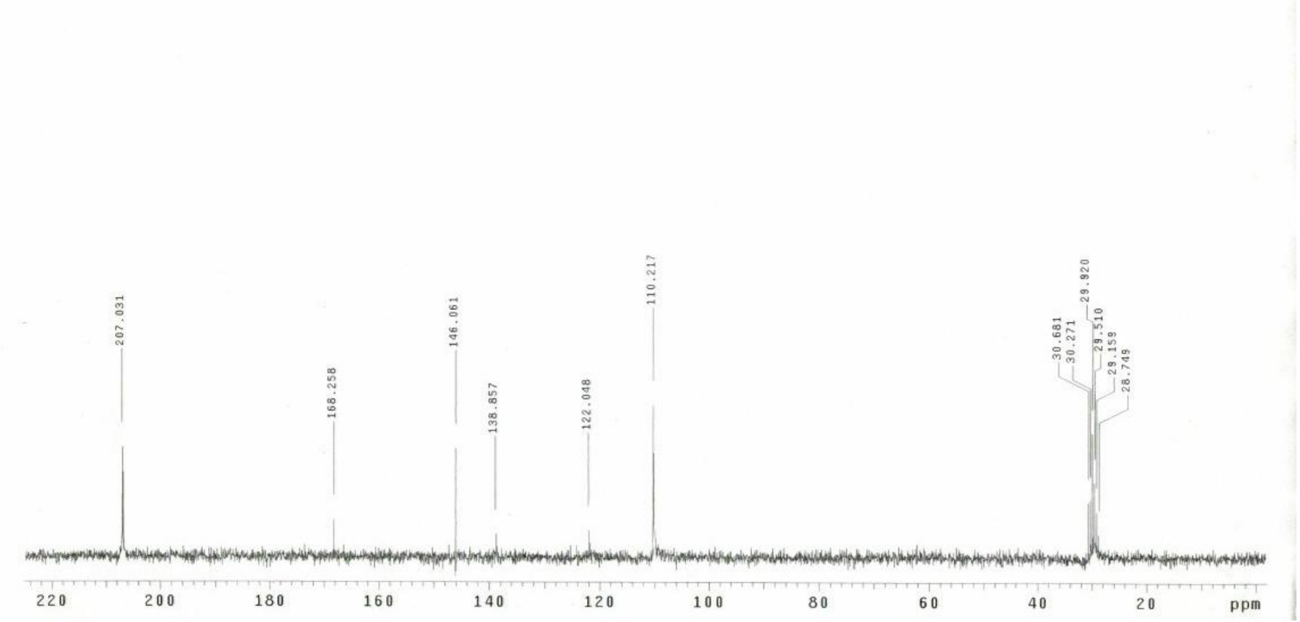

Figure S3. NMR ^{13}C spectrum of gallic acid [(100 MHz, ACETONE-d_6), δ (ppm)].

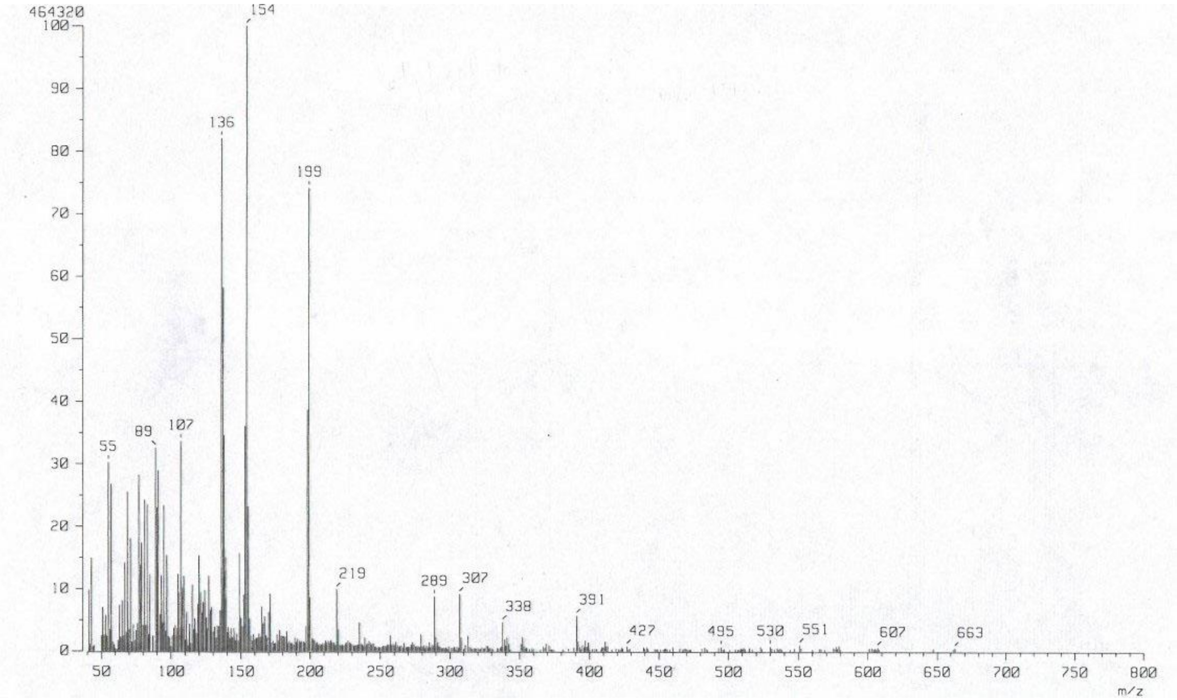

Figure S4. Mass spectrum of ethyl gallate

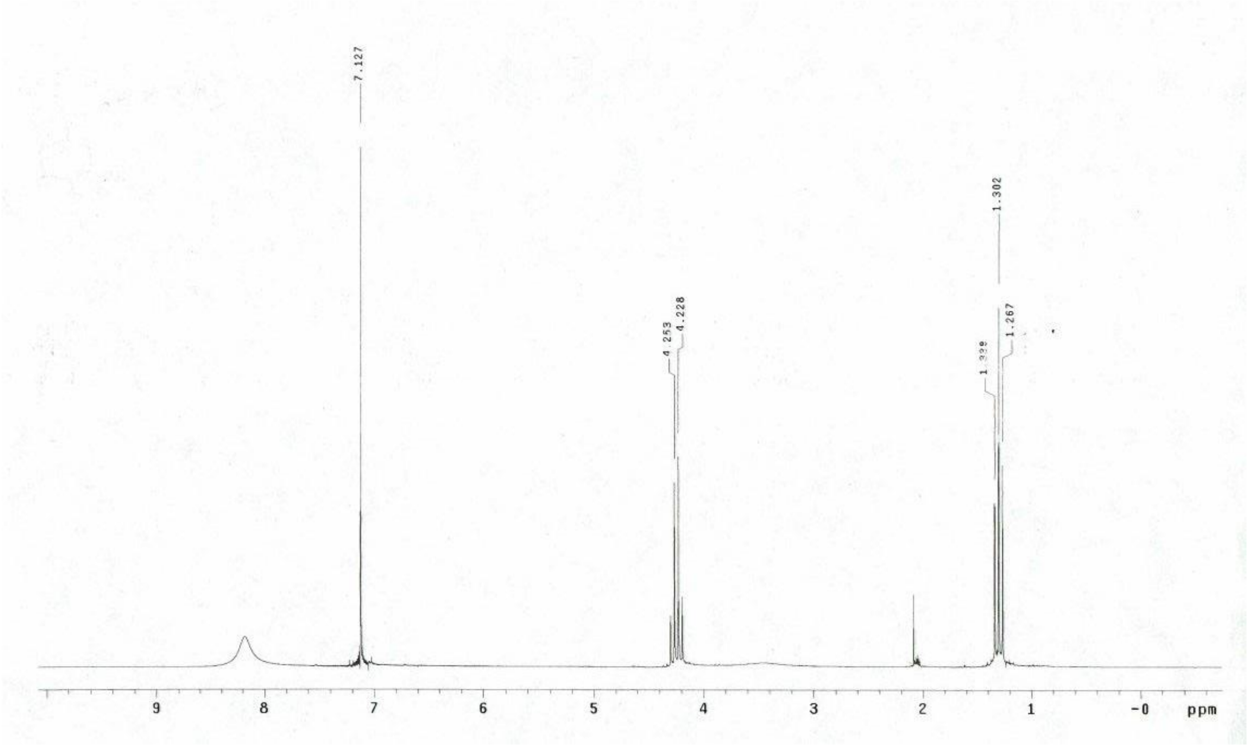

Figure S5. NMR ^{1}H spectrum of ethyl gallate [(400 MHz, ACETONE-d_6), δ (ppm)].

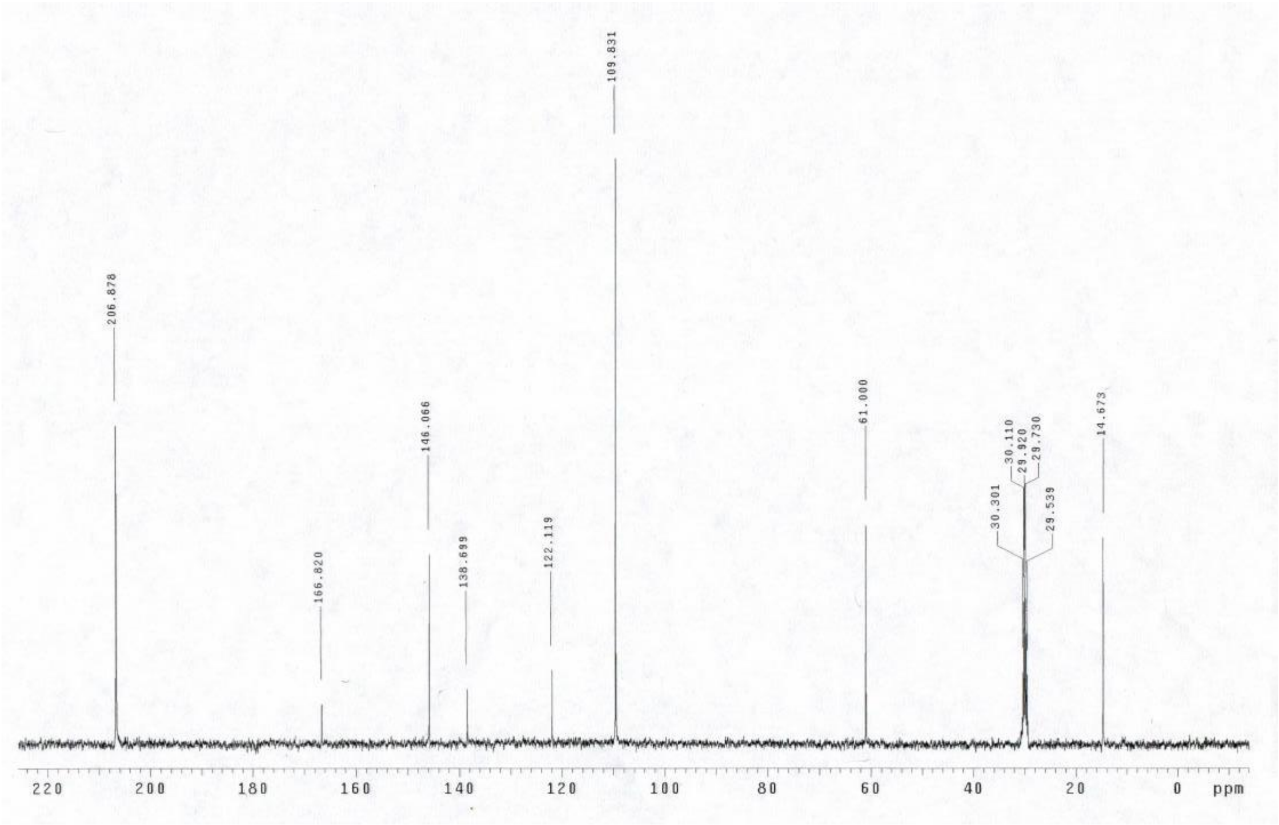

Figure S6. NMR ^{13}C spectrum of ethyl gallate [(100 MHz, ACETONE-d_6), δ (ppm)].

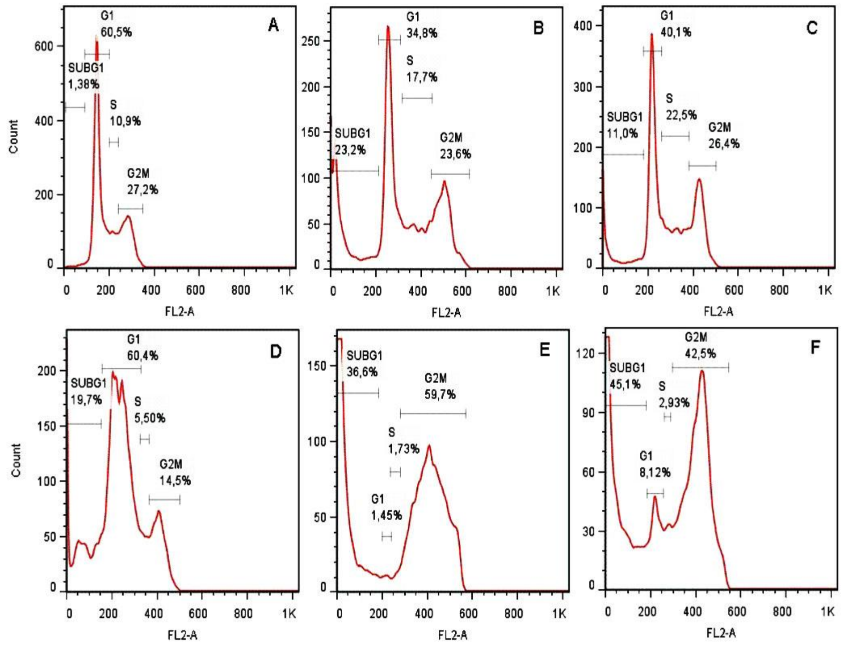

Figure S7. Effect of *C. coriaria* extract and isolated compounds on cell cycle in PC3 cell line of prostate cancer. (A) Negative control; (B) *C. coriaria* extract; (C) Gallic acid; (D) Ethyl gallate; (E) Tannic acid; (F) Podophillotoxin 0.005μM (positive control).

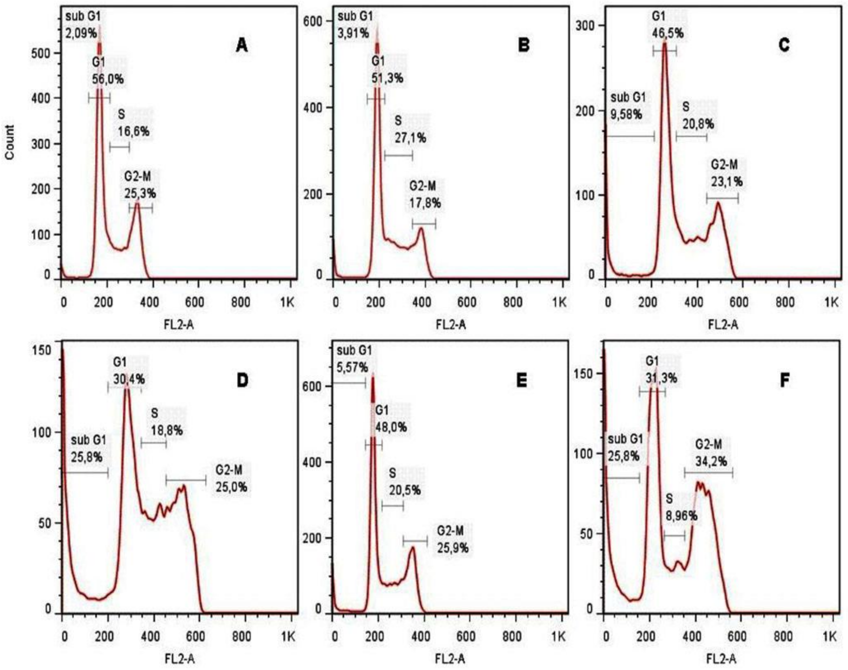

Figure S8. Effect of *C. coriaria* extract and isolated compounds on cell cycle in Hep3B cell line of hepatocellular carcinoma. (A) Negative control; (B) *C. coriaria* extract; (C) Gallic acid; (D) Ethyl gallate; (E) Tannic acid; (F) Podophillotoxin 0.005μM (positive control).

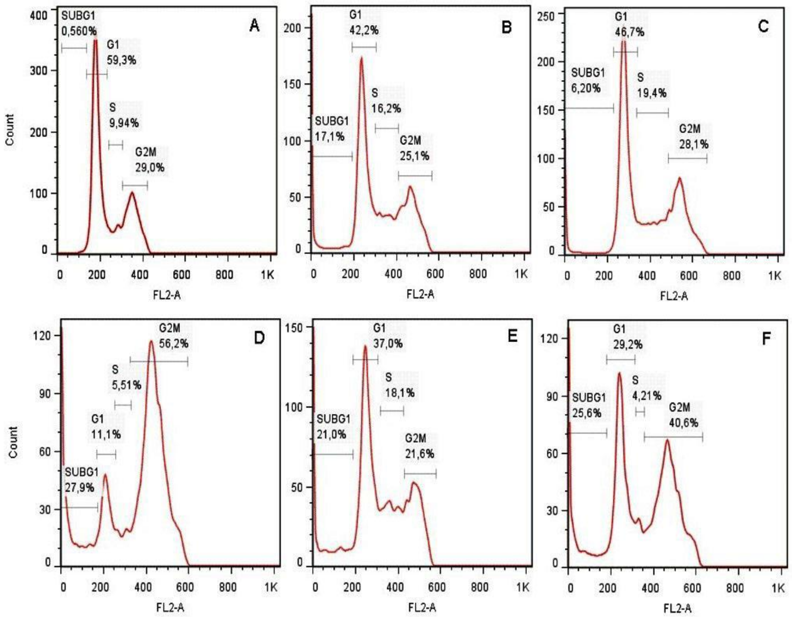

Figure S9. Effect of *C. coriaria* extract and isolated compounds on cell cycle in HepG2 cell line of hepatocellular carcinoma. (A) Negative control; (B) *C. coriaria* extract; (C) Gallic acid; (D) Ethyl gallate; (E)Tannic acid; (F) Podophillotoxin 0.005µM (positive control).

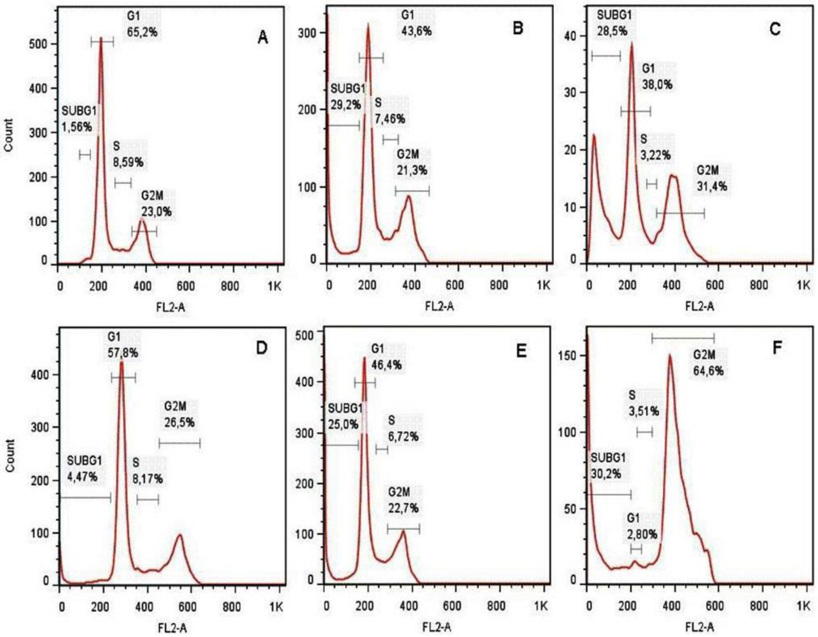

Figure S10. Effect of *C. coriaria* extract and isolated compounds on cell cycle in HeLa cell line of cervical cancer. (A) Negative control; (B) *C. coriaria* extract; (C) Gallic acid; (D) Ethyl gallate; (E) Tannic acid; (F) Podophillotoxin 0.005µM (positive control).

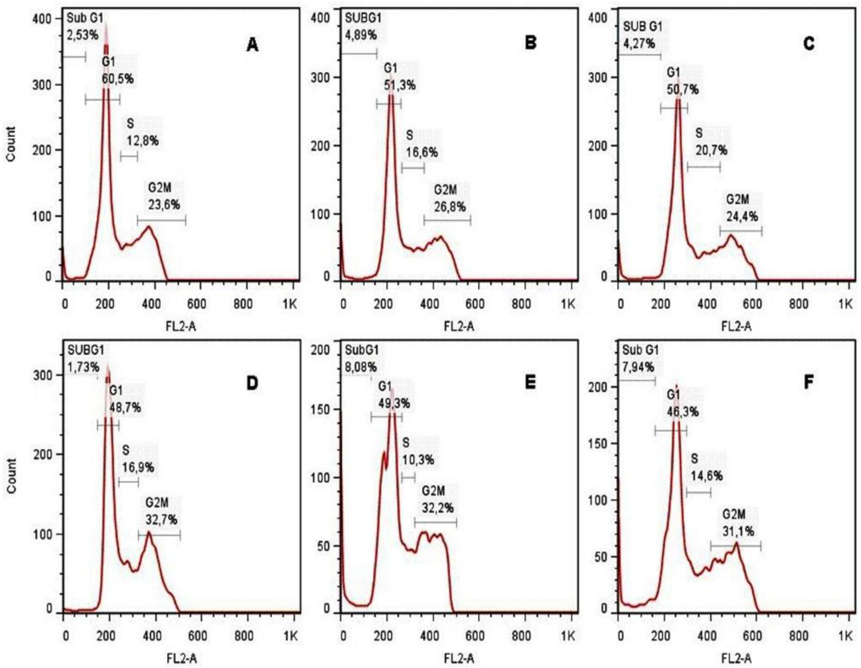

Figure S11. Effect of *C. coriaria* extract and isolated compounds on cell cycle in CaS ki cell line of cervical cancer. (A) Negative control; (B) *C. coriaria* extract; (C) Gallic acid; (D) Ethyl gallate; (E) Tannic acid; (F) Podophillotoxin 0.005μM (positive control).

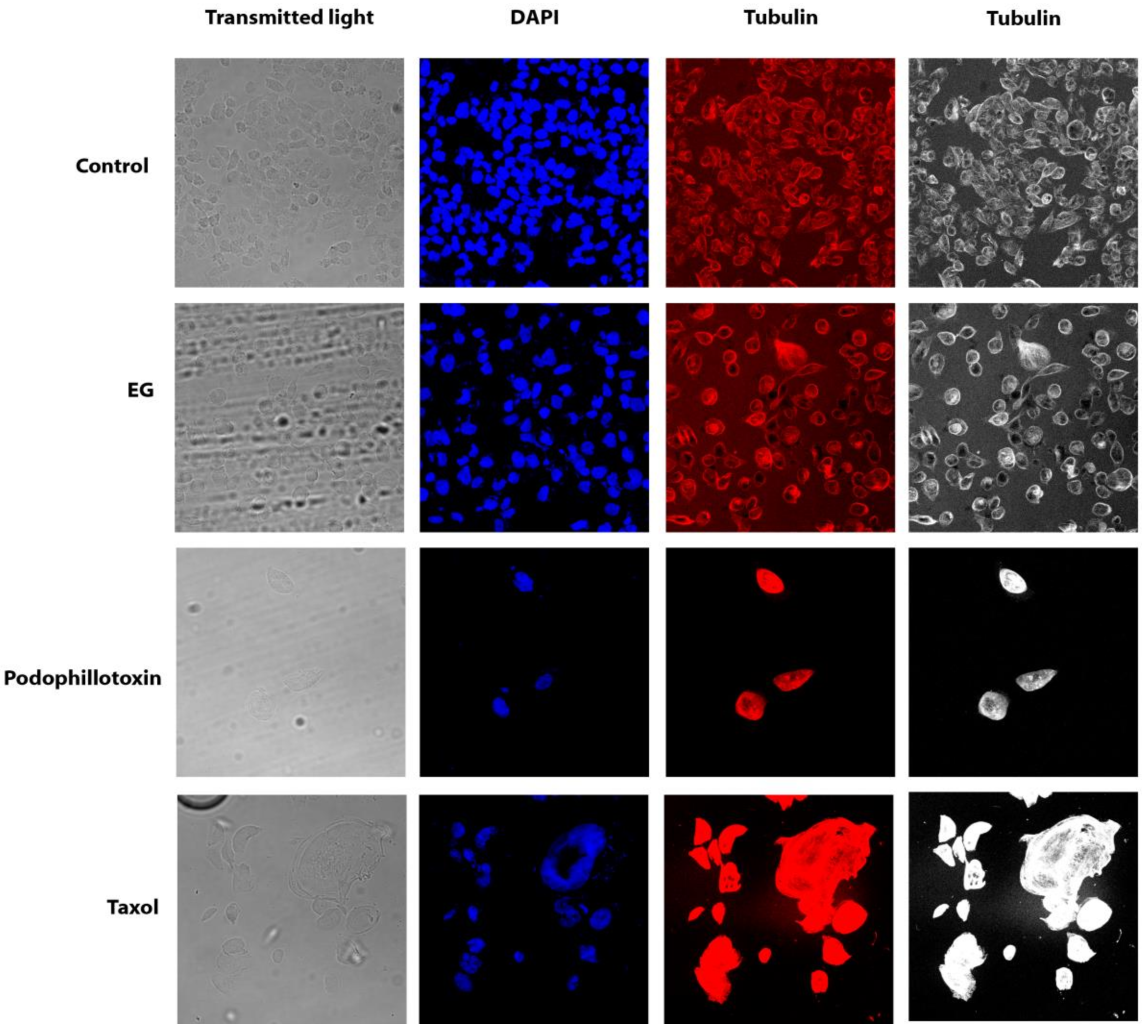

Figure S12. Effect of EG microtubules in PC3 cells by confocal microscopy. Podophillotoxin (Microtubules destabilizing agent) and Taxol (Microtubules stabilizing agent).

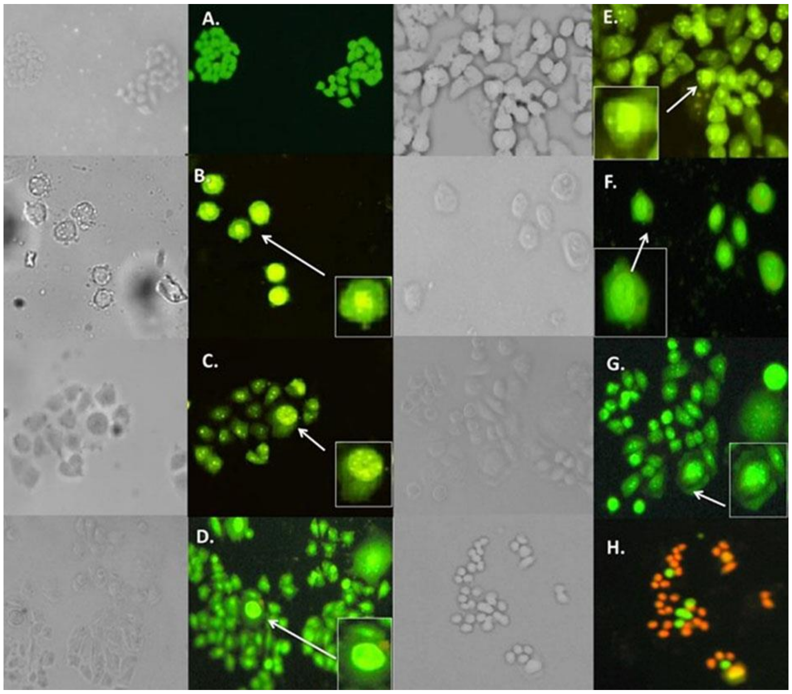

Figure S13. Effect of extract *C.coriaria* and isolated compounds on cell death by epifluorescence microscopy in PC3 cell line of prostate cancer. (A) Negative control; (B) *C. coriaria* extract; (C) Gallic acid; (D) Ethyl gallate; (E) Tannic acid; (F) Podophillotoxin 0.005µM (positive control); (G) H_2O_2 apoptosis positive control; (H) Necrosis control.

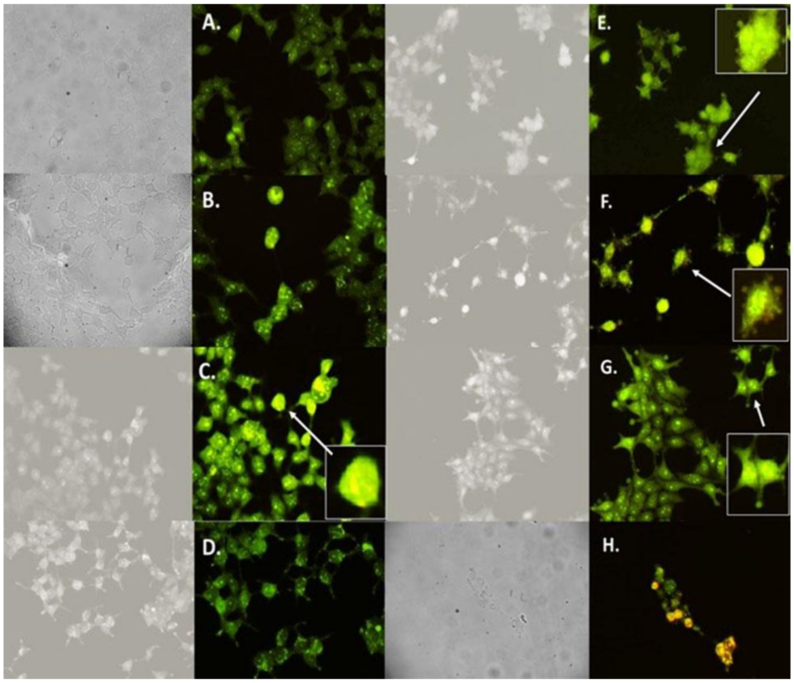

Figure S14. Effect of extract *C.coriaria* and isolated compounds on cell death by epifluorescence microscopy in HepG2 cell line of hepatocellular carcinoma. (A) Negative control; (B) *C. coriaria* extract; (C) Gallic acid; (D) Ethyl gallate; (E) Tannic acid; (F) Podophillotoxin 0.005µM (positive control); (G) H_2O_2 apoptosis positive control; (H) Necrosis control.

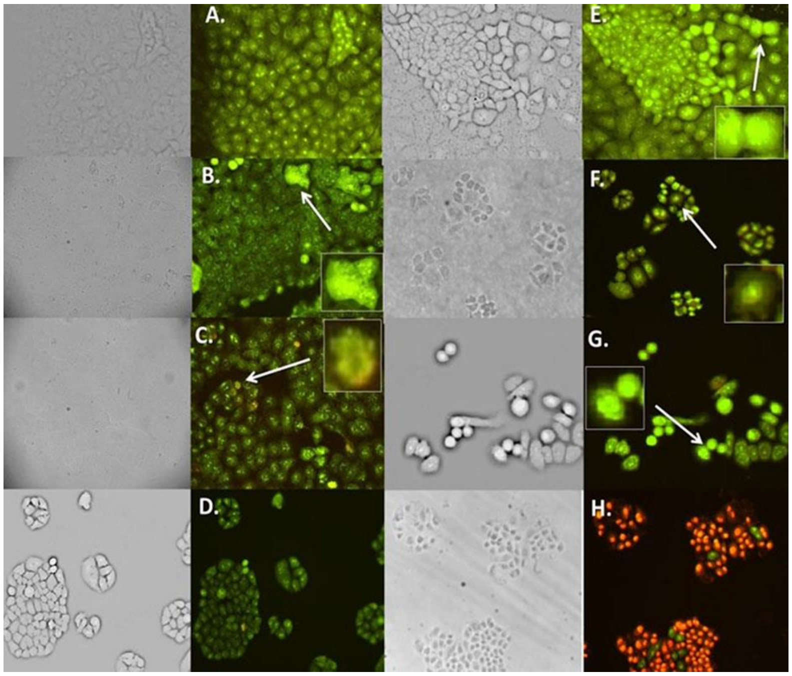

Figure S15. Effect of extract *C.coriaria* and isolated compounds on cell death by epifluorescence microscopy in HeLa cell line of cervical cancer. (A) Negative Control; (B) *C. coriaria* extract; (C) Gallic acid; (D) Ethyl gallate; (E) Tannic acid; (F) Podophillotoxin 0.005μM (positive control); (G) H_2O_2 apoptosis positive control; (H) Necrosis control.

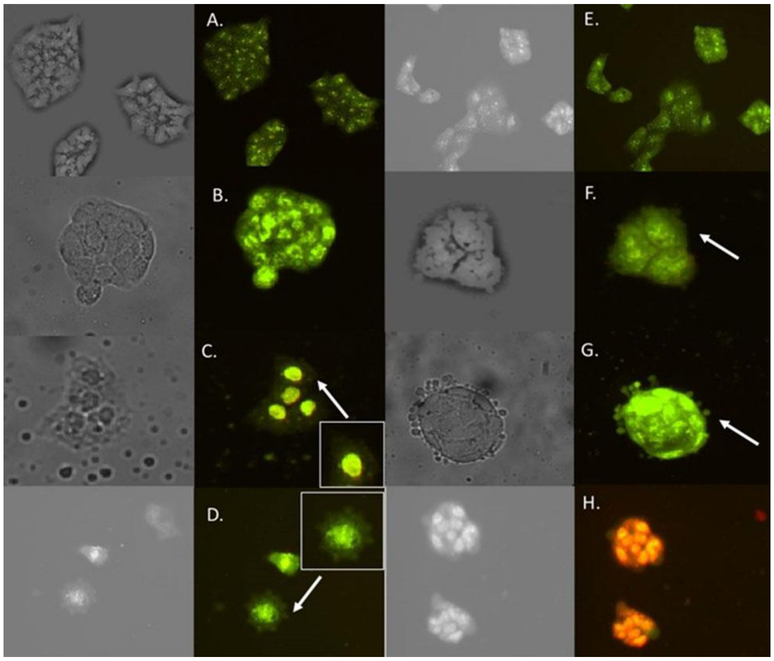

Figure S16. Effect of extract *C.coriaria* and isolated compounds on cell death by epifluorescence microscopy in CaS ki cell line of cervical cancer. (A) Negative Control; (B) *C. coriaria* extract; (C) Gallic acid; (D) Ethyl gallate; (E) Tannic acid; (F) Podophillotoxin 0.005μM (positive control); (G) H_2O_2 apoptosis positive control; (H) Necrosis control.
